# Remote smartphone-based spoken language screening predicts clinical markers in Huntington’s disease

**DOI:** 10.1007/s00702-026-03173-5

**Published:** 2026-05-14

**Authors:** Martin Šubert, Tomáš Kouba, Tereza Tykalová, Olga Klempířová, Alzbeta Mühlbäck, Jiří Klempíř, Jan Rusz

**Affiliations:** 1 Department of Circuit Theory, Faculty of Electrical Engineering, Czech Technical University in Prague, Technická 2, Praha 6, 160 00 Czech Republic; 2 Department of Neurology, Centre of Clinical Neuroscience, First Faculty of Medicine, Charles University and General University Hospital, Prague, Czech Republic; 3 Huntington Center South, kbo-isar-Amper-Klinikum, Taufkirchen (Vils), Germany; 4 Department of Neurology, University Hospital Ulm, Ulm University, Ulm, Germany

**Keywords:** Linguistic features, Natural language processing, Speech, Machine learning, Digital biomarker

## Abstract

Assessment and monitoring of Huntington’s disease (HD) symptoms remain limited to infrequent, clinic-based evaluations. We evaluated whether fully automated linguistic analysis of speech tasks recorded via smartphone can remotely capture core clinical markers of HD severity. In this cross-sectional multicenter study across Czech and German sites, 53 participants, including 30 HD (9 pre-symptomatic, 3 prodromal, and 18 manifest) and 23 healthy controls, completed a smartphone-based speech assessment, including spontaneous monologue and fairy tale retelling for 7 consecutive days. Recordings were automatically transcribed and analyzed using natural language processing to derive 3 lexical and 3 syntactic features. Predictive models for clinical outcomes based on the Unified Huntington’s Disease Rating Scale and cognitive scales were built using multivariate linear regression with cross-validation. Linguistic features predicted HD severity across multiple domains with high predictive performance, explaining up to 57% of the variance in cognitive performance, 63% in motor impairment, and 59% in functional capacity. A median of 6 days for monologue and 3 for retelling was sufficient to reach 90% of maximal predictive performance. Compared to controls, HD participants showed reduced vocabulary range and increased phrase repetition in both tasks (*p* < 0.05), with additional monologue-specific deficits in sentence length (*p* = 0.018) and syntactic complexity (*p* = 0.004). Fully automated analysis of smartphone-based language assessment can remotely quantify cognitive, motor, and functional impairment in HD, offering a scalable, low-burden digital biomarker for clinical trials and decentralized monitoring.

**Supplementary Information:**

The online version contains supplementary material available at https://doi.org/10.1007/s00702-026-03173-5.

## Introduction

Huntington’s disease (HD) is a severe inherited neurodegenerative disease caused by an abnormal expansion of cytosine-adenine-guanine (CAG) repeats in the huntingtin (HTT) gene (MacDonald et al. 1993), leading to progressive motor, cognitive, and behavioral impairments. Although recent therapeutic efforts target the underlying pathophysiology, effective disease-modifying interventions remain under development (Ross et al. 2019; Tabrizi et al. 2022). Sensitive and scalable biomarkers are thus essential for early detection, precise monitoring of disease progression, and evaluation of emerging treatments. In current clinical practice, clinical symptoms are monitored using the Unified Huntington’s Disease Rating Scale (UHDRS) (1996), the worldwide reference scale for evaluating HD. However, the assessment requires a high level of expertise and is often affected by limited interrater reliability among physicians (Hogarth et al. 2005; Mestre et al. 2018; Winder et al. 2018). Moreover, reliance on in-person testing is time-consuming, costly, and insensitive to subtle or short-term changes, underscoring the need for objective, low-burden digital biomarkers suitable for remote monitoring.

Language production is a complex, integrative function that engages cortical–subcortical circuits, which are particularly vulnerable in HD, including the striatum and prefrontal regions (Murray 2000; Teichmann et al. 2008; Puig-Davi et al. 2025). Subtle linguistic changes, such as reduced lexical diversity, increased repetition, and simplified syntactic structures, may therefore precede motor symptoms and reflect early degeneration across cognitive and motor domains, as demonstrated in previous studies (Chan et al. 2019; Tovar et al. 2020; Nunes et al. 2024). However, most prior work relied on laboratory-based assessment and partially manual annotation, limiting scalability and feasibility for frequent monitoring (Chan et al. 2019; Tovar et al. 2020; Nunes et al. 2024). Recent advances in automatic speech recognition (ASR) and natural language processing (NLP) now enable fully automated linguistic analysis directly from raw speech without human intervention, even on devices with low-quality microphones such as smartphones. Yet, whether fully automated, smartphone-based analysis of speech can capture clinically meaningful variation in main HD symptoms remains unknown. To provide further insights into smartphone-based monitoring, it is also necessary to determine the minimum sample duration required to achieve optimal screening performance with smartphone-captured speech, and to assess how different spontaneous speech tasks used for discourse analysis may pose difficulties for patients with HD in a real-world setting.

Therefore, in this multicenter study using two different languages, we aimed to determine whether fully automated linguistic analysis can predict the standard clinical measures from the UHDRS and other relevant clinical scales in HD gene carriers.

## Methods

### Standard protocol approvals, registrations, and patient consents

The study obtained approval from the local responsible ethical committees on human experimentation and was conducted in accordance with the ethical standards established in the 1964 Declaration of Helsinki. All participants provided written, informed consent to the neurological examination and recording procedure.

### Study design

We investigated two languages, Czech and German, in which Czech native speakers were enrolled at Charles University and the General University Hospital, Prague, Czech Republic, while German native speakers were recruited from Huntington’s centers at the kbo-Isar-Amper-Klinikum, Taufkirchen, and the Department of Neurology at Ulm University, Germany. All participants in the HD group had genetically confirmed disease, defined by ≥ 36 CAG repeats in one HTT allele. Exclusion criteria included a history of communication disorders or significant neurological conditions unrelated to HD. Healthy controls were excluded if they had a history of communication or neurological disorders.

During on-site clinic visits, HD participants underwent standardized clinical assessments from the UHDRS battery, covering the main disease-affected domains in HD. These included total functional capacity (UHDRS-TFC) (1996), total motor score (UHDRS-TMS) (1996), and cognitive performance, evaluated using the Symbol Digit Modalities Test (SDMT) (Strober et al. 2019), lexical and categorical verbal fluency (VF) (1996), Stroop Tests (ST) (1996), Trail Making Tests parts A and B (TMT-A and TMT-B) (Reitan 1958), and the Mini-Mental State Examination (MMSE) (Folstein et al. 1975). Participants with HD were evaluated using the UHDRS Diagnostic Confidence Level (DCL) (Ross et al. 2019), which reflects the clinician’s certainty of the diagnosis based on the presence and severity of motor signs (Oosterloo et al. 2021).

### Smartphone-based speech assessment

During the initial clinic visit, participants were provided with a smartphone HONOR 9X Lite (Shenzhen Zhixin New Information Technology, Shenzhen, China) running Android 9. The device was preloaded with a custom-developed application (Kouba 2025) consisting of 5 speech and 5 motor tests. For the purposes of language assessment, two speech tasks were analyzed: monologue and retelling. In the monologue task, the application prompted participants to answer three sequential questions, designed to elicit at least 90 s of continuous speech. To sustain engagement and encourage longer responses, 7 distinct sets of questions were designed (Table S1) and rotated each day. In the retelling task, participants listened to a narrated fairy tale lasting approximately 90 s and were then instructed to retell the story in their own words, maintaining comparable detail and duration. To minimize learning effects, the 7 most common fairy tales were selected for both Czech and German languages separately (Table S2) and rotated daily. The average length of the reference fairy tales was 249 (SD 24) words.

The explanation of the application functions and the first speech assessment were conducted in the clinic under clinician supervision to ensure that participants fully understood the procedure. This was followed by additional self-administered speech assessments, completed remotely by participants at home during 6 consecutive days.

### Speech transcription

All recordings were resampled to 16 kHz and transcribed using the Whisper automatic speech recognition system (Radford et al. 2022), utilizing the latest multilingual large-v3 model. To enhance transcription accuracy and reduce hallucination errors (instances in which the system inserts words not actually spoken, often due to background noise, atypical speech patterns, or model limitations) we implemented an algorithm to identify and remove such artifacts. Details on transcription accuracy of this system have been published previously (Subert et al. 2025a).

### Language analysis

**Fig. 1:**
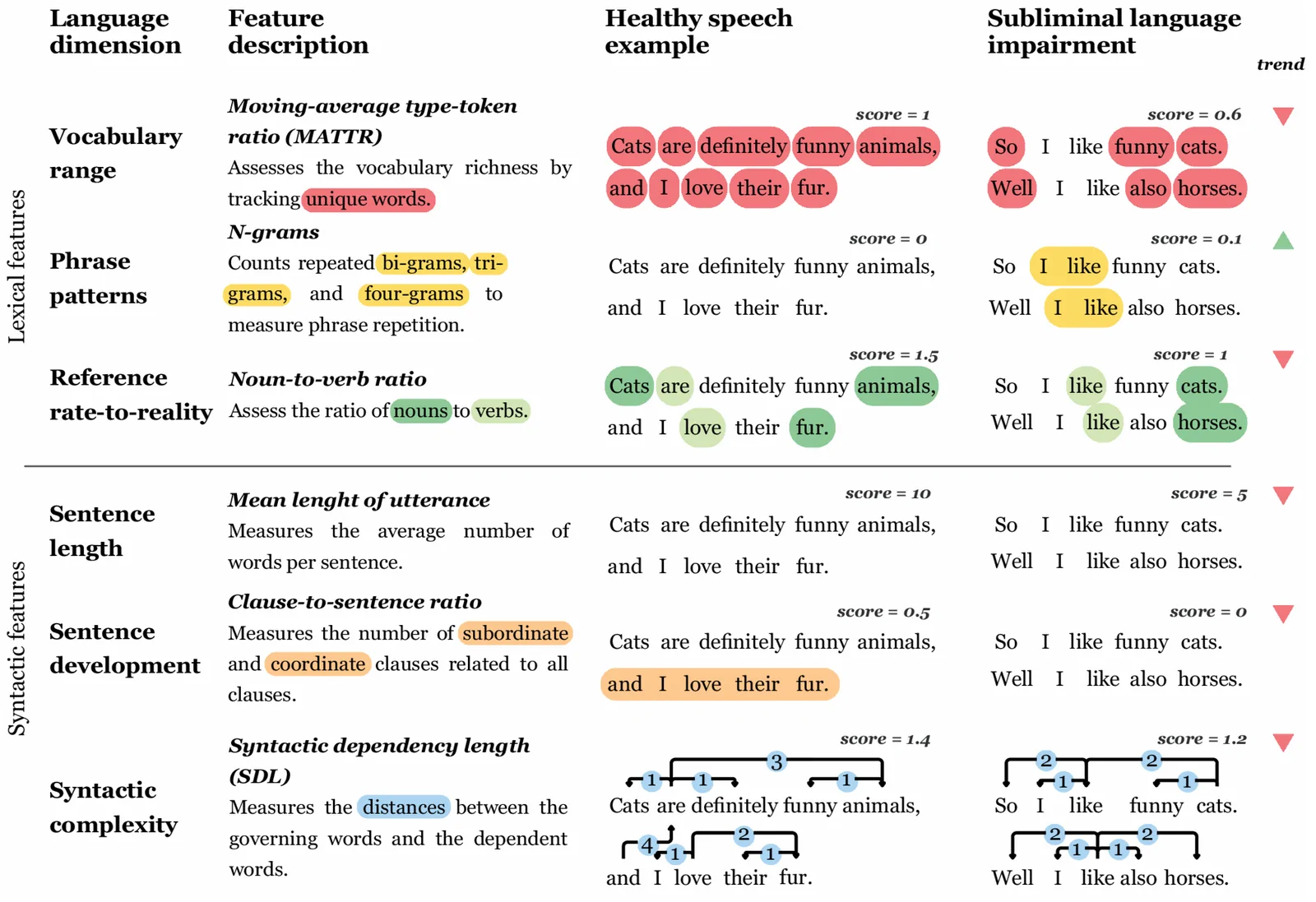
Description of linguistic characteristics

The transcripts were linguistically analyzed and annotated using natural language processing toolkit stanza (Qi et al. 2020), which extracts lexical and syntactic information essential for subsequent computation of linguistic features. The selection of features was based on the following criteria: (i) previously demonstrated sensitivity to language decline in mild cognitive impairment, consistent with well-established cognitive-linguistic models and supported by robust evidence of language alterations across studies (Taler and Phillips 2008; Beltrami et al. 2018; Mueller et al. 2018), (ii) coverage of distinct aspects of language to capture a complex language profile, and (iii) compatibility with fully automated computation using available techniques. Based on these criteria, we selected 3 lexical and 3 syntactic features (Fig. 1). The selection of features is consistent with previously reported HD-related impairments, including reduced lexical diversity, increased repetition, and simplified syntactic structures (Hinzen et al. 2018; Tovar et al. 2020). Limiting the number of features, we aimed to decrease the probability of Type I error.

In the lexical features, *vocabulary range* was quantified using the moving-average type-token ratio (MATTR) (Covington and McFall 2010), which estimates lexical diversity by computing the proportion of unique words within a sliding window across the transcript and averaging across windows. The window size was set to 34 words, based on the constraints of our dataset and prior recommendations for evaluating patients’ vocabulary (Covington and McFall 2010). *Phrase patterns* were quantified using normalized counts of repeated bi-, tri-, and four-grams (Šubert et al. 2022), reflecting the tendency to reuse fixed word sequences rather than generate novel phrasing. *Reference rate to reality* was calculated as a ratio of nouns to verbs, reflecting the tendency toward object-focused (nouns) or action-oriented (verbs) language.

Regarding the syntactic features, *sentence length* was calculated using mean length of utterance, defined as the average number of words per utterance (Subert et al. 2025a). An utterance was defined as sentence-like units corresponding to sequences of words terminated by a period. Punctuation was assigned automatically by the Whisper system using acoustic and linguistic cues, ensuring consistent and reproducible segmentation. *Sentence development* was assessed using clause-to-sentence ratio (Subert et al. 2025b), which is the ratio of a total number of subordinate and coordinate clauses to the total number of sentences. This measure assesses the tendency toward developing sentences into more complex structures. *Syntactic complexity* was assessed using syntactic dependency length (Subert et al. 2025b), calculated as the number of words intervening between a governing element (e.g., a verb) and its dependent (e.g., a subject or object) for each word in a sentence. Each value is normalized by the number of words in the sentence, and the final score is obtained by averaging these normalized values.

### Statistical analysis

Linguistic features were extracted separately for each task (monologue and retelling) on each day of recording. Based on analysis of covariance (ANCOVA), both for monologue and retelling task, we found no significant interaction between group and language in any of linguistic features (Fig. S1); analyses were based on mean linguistic feature values across all 7 recording days and were adjusted for age and sex as covariates. To evaluate the effect of repeated recordings, feature values for each day were computed as the cumulative mean of all preceding days, such that day 1 reflects that day’s recording, day 2 reflects the mean of days 1 and 2, and so on. The primary outcome was to assess whether language characteristics could predict standard clinical markers. We applied multivariate linear regression with leave-one-out cross-validation, allowing the model to include both linguistic features and demographic variables (age, sex, and Czech/German language). To account for heterogeneity in disease stage, an additional regression analysis incorporated also DCL as a predictor.

Feature selection was performed using an exhaustive best-subset approach, evaluating all possible combinations of predictors up to a maximum of four predictors. The final model was selected based on the highest cross-validated R^2^ obtained via leave-one-out cross-validation. To reduce the risk of overfitting, model complexity was constrained to a maximum of four predictors in total, including both linguistic features and demographic variables (or DCL), corresponding to approximately 7.5 subjects per predictor, consistent with recommended ratios of 5–10 observations per predictor (Austin and Steyerberg 2015; Jenkins and Quintana-Ascencio 2020). A priori selection of features with unique language-domain relevance minimized redundancy, and cross-validation further mitigated overfitting.

To determine the minimal number of recording days required for reliable predictions, we defined a threshold corresponding to 90% of the maximum achievable R^2^.

Group differences in linguistic features were assessed using ANCOVA with age, sex, and language as covariates. To account for multiple comparisons across the set of linguistic features, p-values were adjusted using the Benjamini-Hochberg procedure to control the false discovery rate; adjusted p-values (q-values) below α = 0.05 were considered statistically significant.

## Results

### Participants

A total of 53 participants were enrolled, including 30 individuals with HD (14 males; 21 Czech and 9 German) and 23 healthy controls (11 males; 17 Czech and 6 German), matched for age (*p* = 0.69) and sex (*p* = 0.94). The mean age was 47.1 years (SD = 9.8) in the HD group and 46.0 years (SD = 11.2) in the control group. Within the HD cohort, UHDRS-DCL staging classified 7 participants as normal (DCL = 0), 2 with non-specific motor abnormalities (< 50% confidence, DCL = 1), 3 with motor abnormalities that may be signs of HD (50–89% confidence, DCL = 2), and 18 with motor abnormalities that are unequivocal signs of HD (≥ 99% confidence, DCL = 4) (Table 1).

**Table 1:** Demographic and clinical data

	Huntington’s disease (*n* = 30)
Age (y)	47.1/9.8 (30–65)
Sex (male)	14 (47%)
Cytosine-Adenine-Guanine (CAG)	43.6/3.4 (39–53)
UHDRS – Diagnostic Confidence Level	2.8/1.7 (0–4)
UHDRS – Total Motor Score	16.1/14.7 (0–51)
UHDRS – Total Functional Capacity	11.0/2.3 (6–13)
Symbol Digit Modalities Test	38.7/17.1 (10–70)
Lexical verbal fluency	36.9/16.8 (10–65)
Categorical verbal fluency	19.5/7.0 (7–34)
Stroop Color Test	59.3/18.4 (31–90)
Stroop Word Test	80.1/22.8 (40–123)
Stroop Interference Test	35.3/14.3 (17–67)
Trail Making Test A	36.8/21.5 (12–91)
Trail Making Test B	76.6/64.1 (13–240)
Mini-Mental State Examination	27.8/2.8 (20–30)

Data are presented as mean/standard deviation (range) or as number (%), as appropriate

*UHDRS *Unified Huntington’s Disease Rating Scale

### Predicting clinical markers from language characteristics

The mean word count per recording was 231 (SD 124) for monologues and 186 (SD 73) for retellings. Models including only demographic variables (age, sex, and Czech/German language) showed limited explanatory power across all clinical scales (R^2^ < 0.16).

**Fig. 2:**
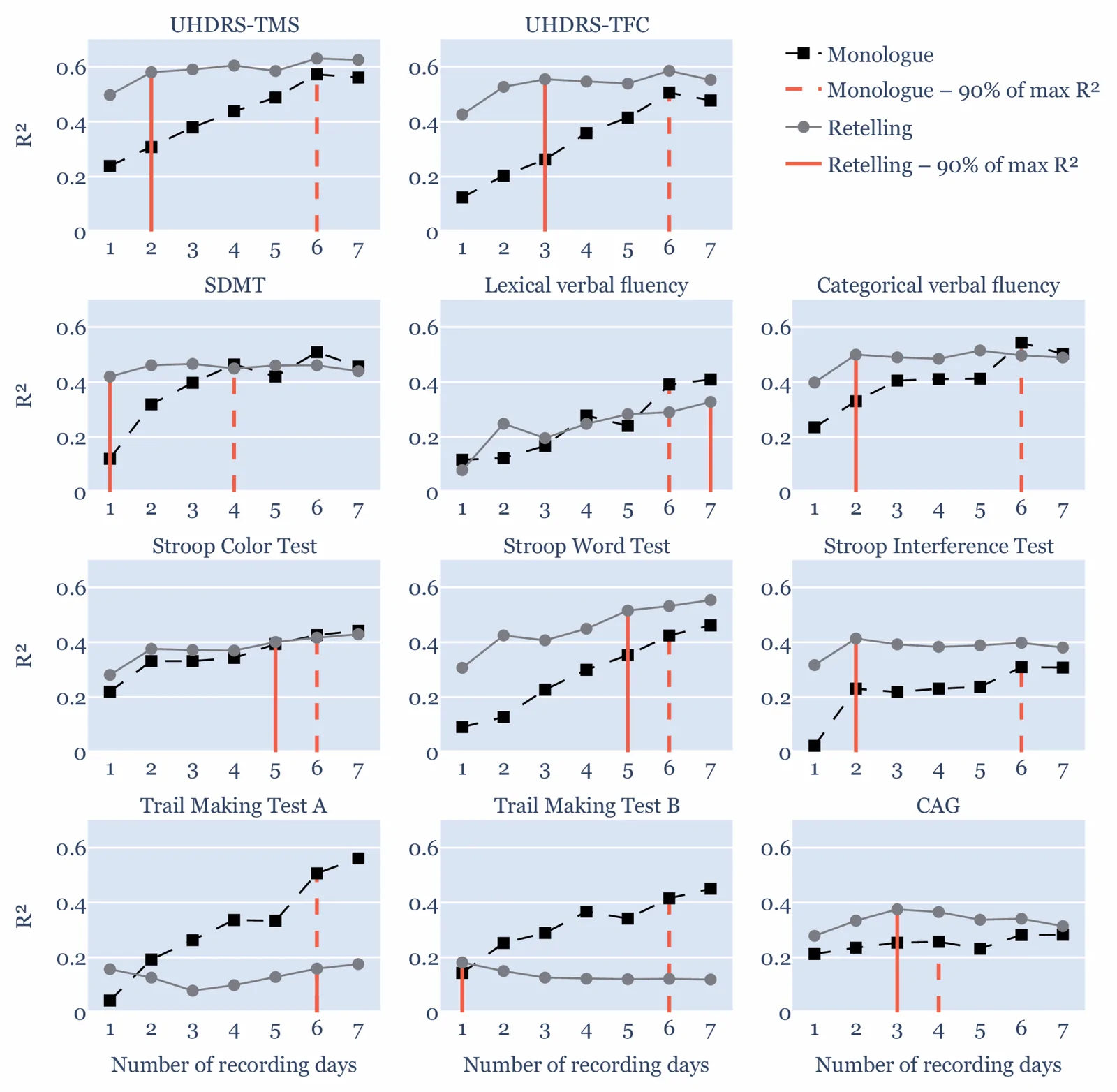
Stabilization of predictive performance of linguistic features across repeated smartphone recordings. *UHDRS-TMS* Unified Huntington’s Disease Rating Scale Total Motor Score, *UHDRS-TFC*  Unified Huntington’s Disease Rating Scale Total Functional Capacity, *SDMT*  Symbol Digit Modalities Test, *CAG*  cytosine-adenine-guanine

In the monologue task, linguistic features predicted motor impairment (UHDRS-TMS; R^2^ = 0.57, *p* < 0.001) and functional capacity (UHDRS-TFC; R^2^ = 0.51, *p* < 0.001) (Table 2). Cognitive measures of processing speed and executive function, including SDMT, VF, ST, and TMT, were predicted with moderate to strong predictive performance (R^2^ = 0.31–0.57, *p* ≤ 0.002). CAG repeat length was modestly predicted (R^2^ = 0.28, *p* = 0.002), whereas predictive performance for MMSE was low (R^2^ < 0.20). Stability thresholds were reached after 6 days of recordings, except for SDMT and CAG, which stabilized after 4 days (Fig. 2).

**Table 2:** Predictive performance of linguistic features for clinical markers in monologue tasks

Clinical scale	Predictors selected through feature selection (β, *p*-value)	*R* ^2^	*p*-value
UHDRS-TMS	Vocabulary range (β=-0.17, *p* = 0.21) Sentence length (β=-0.48, *p* = 0.024) Syntactic complexity (β=-0.37, *p* = 0.21) Language (β = 0.60, *p* = 0.020)	0.57	< 0.001
UHDRS-TFC	Vocabulary range (β = 0.50, *p* < 0.001) Phrase patterns (β = 0.27, *p* = 0.061) Sentence length (β = 0.63, *p* < 0.001) Sex (β = 0.22, *p* = 0.11)	0.51	< 0.001
SDMT	Phrase patterns (β=-0.28, *p* = 0.035) Sentence length (β = 1.43, *p* < 0.001) Sentence development (β=-1.12, *p* < 0.001) Language (β=-0.45, *p* = 0.003)	0.51	< 0.001
Lexical verbal fluency	Phrase patterns (β=-0.20, *p* = 0.19) Sentence length (β = 1.50, *p* < 0.001) Sentence development (β=-0.82, *p* = 0.010) Syntactic complexity (β=-0.86, *p* < 0.001)	0.41	< 0.001
Categorical verbal fluency	Sentence length (β = 1.21, *p* < 0.001) Sentence development (β=-0.62, *p* = 0.013) Sex (β = 0.20, *p* = 0.11) Language (β=-0.37, *p* = 0.010)	0.54	< 0.001
Stroop Color Test	Vocabulary range (β = 0.46, *p* = 0.007) Reference rate to reality (β=-0.32, *p* = 0.051) Sentence length (β = 0.77, *p* < 0.001) Syntactic complexity (β=-0.28, *p* = 0.090)	0.44	< 0.001
Stroop Word Test	Vocabulary range (β = 0.28, *p* = 0.095) Reference rate to reality (β=-0.15, *p* = 0.32) Sentence length (β = 1.11, *p* < 0.001) Syntactic complexity (β=-0.65, *p* = 0.011)	0.46	< 0.001
Stroop Interference Test	Phrase patterns (β=-0.30, *p* = 0.070) Sentence length (β = 1.14, *p* < 0.001) Sentence development (β=-0.81, *p* = 0.011) Language (β=-0.27, *p* = 0.11)	0.31	0.002
Trail Making Test A	Phrase patterns (β = 0.36, *p* = 0.009) Sentence length (β=-1.29, *p* < 0.001) Sentence development (β = 1.13, *p* < 0.001)	0.57	< 0.001
Trail Making Test B	Phrase patterns (β = 0.32, *p* = 0.031) Sentence length (β=-0.93, *p* = 0.013) Sentence development (β = 0.96, *p* = 0.003) Syntactic complexity (β=-0.23, *p* = 0.26)	0.47	< 0.001
CAG	Sentence length (β=-0.30, *p* = 0.19) Syntactic complexity (β=-0.29, *p* = 0.39) Age (β=-0.41, *p* = 0.010) Language (β = 0.38, *p* = 0.18)	0.28	0.002

*β* standardized beta value of individual predictors, *UHDRS-TMS* Unified Huntington’s Disease Rating Scale Total Motor Score, *UHDRS-TFC* Unified Huntington’s Disease Rating Scale Total Functional Capacity, *SDMT* Symbol Digit Modalities Test, *CAG* cytosine-adenine-guanine

In the retelling task, the highest predictive performance was observed for motor decline (UHDRS-TMS; R^2^ = 0.63, *p* < 0.001) and functional capacity (UHDRS-TFC; R^2^ = 0.59, *p* < 0.001) (Table 3). Cognitive measure of processing speed and executive function were predicted with moderate predictive performance (R^2^ = 0.18–0.55, *p* ≤ 0.048). CAG repeat length was also moderately predicted (R^2^ = 0.38, *p* < 0.001), while MMSE prediction again remained low (R^2^ < 0.20). Stability thresholds varied from 1  to 7 days.

**Table 3:** Predictive performance of linguistic features for clinical markers in retelling task

Clinical scale	Predictors selected through feature selection (β, *p*-value)	*R* ^2^	*p*-value
UHDRS-TMS	Vocabulary range (β=-0.30, *p* = 0.048) Phrase patterns (β = 0.55, *p* = 0.002) Reference rate to reality (β=-0.22, *p* = 0.096) Sentence development (β=-0.28, *p* = 0.045)	0.63	< 0.001
UHDRS-TFC	Vocabulary range (β = 0.24, *p* = 0.12) Phrase patterns (β=-0.63, *p* < 0.001) Reference rate to reality (β = 0.32, *p* = 0.016) Sex (β = 0.23, *p* = 0.070)	0.59	< 0.001
SDMT	Vocabulary range (β = 0.55, *p* = 0.003) Phrase patterns (β=-0.49, *p* = 0.03) Age (β=-0.31, *p* = 0.040) Language (β = 0.25, *p* = 0.089)	0.47	< 0.001
Lexical verbal fluency	Vocabulary range (β = 0.60, *p* < 0.001) Sentence development (β = 0.25, *p* = 0.12)	0.33	< 0.001
Categorical verbal fluency	Vocabulary range (β = 0.78, *p* < 0.001) Sentence development (β = 0.47, *p* < 0.01) Language (β = 0.42, *p* < 0.01)	0.52	< 0.001
Stroop Color Test	Phrase patterns (β=-0.77, *p* < 0.001) Reference rate to reality (β = 0.24, *p* = 0.12)	0.43	< 0.001
Stroop Word Test	Phrase patterns (β=-0.90, *p* < 0.001) Reference rate to reality (β = 0.29, *p* = 0.035) Language (β = 0.36, *p* = 0.009)	0.55	< 0.001
Stroop Interference Test	Vocabulary range (β = 0.45, *p* = 0.021) Phrase patterns (β=-0.59, *p* < 0.001) Age (β=-0.22, *p* = 0.17) Language (β = 0.31, *p* = 0.057)	0.41	< 0.001
Trail Making Test A	Phrase patterns (β = 0.57, *p* = 0.005) Reference rate to reality (β=-0.35, *p* = 0.068) Sentence length (β=-0.22, *p* = 0.23) Language (β=-0.48, *p* = 0.010)	0.18	0.005
Trail Making Test B	Vocabulary range (β=-0.36, *p* = 0.073) Syntactic complexity (β=-0.0051, *p* = 0.98) Language (β=-0.54, *p* = 0.012)	0.18	0.048
CAG	Vocabulary range (β=-0.25, *p* = 0.17) Phrase patterns (β = 0.37, *p* = 0.031) Age (β=-0.36, *p* = 0.028)	0.38	< 0.001

*β* standardized beta value of individual predictors, *UHDRS-TMS*  Unified Huntington’s Disease Rating Scale Total Motor Score, *UHDRS-TFC*  Unified Huntington’s Disease Rating Scale Total Functional Capacity, *SDMT*  Symbol Digit Modalities Test, *CAG*  cytosine-adenine-guanine

Models including only demographic variables and DCL achieved predictive performance with R^2^ ranging from 0.13 to 0.58, with the highest performance observed for UHDRS-TMS. Incorporating linguistic features from the monologue task improved predictive performance, reaching an R^2^ of up to 0.76. The largest gains (Δ R^2^ > 0.20) were observed for UHDRS-TFC, SDMT, Lexical VF, the Stroop Test (Word subtest), and TMT-A (Table S3). A similar pattern was observed when linguistic features from the retelling task were included. In this case, models achieved an R^2^ of up to 0.81, with the most substantial improvements (Δ R^2^ > 0.20) observed for UHDRS-TFC, UHDRS-TMS, SDMT, and the Stroop Test (Table S4).

### Group differences using language characteristics

**Fig. 3:**
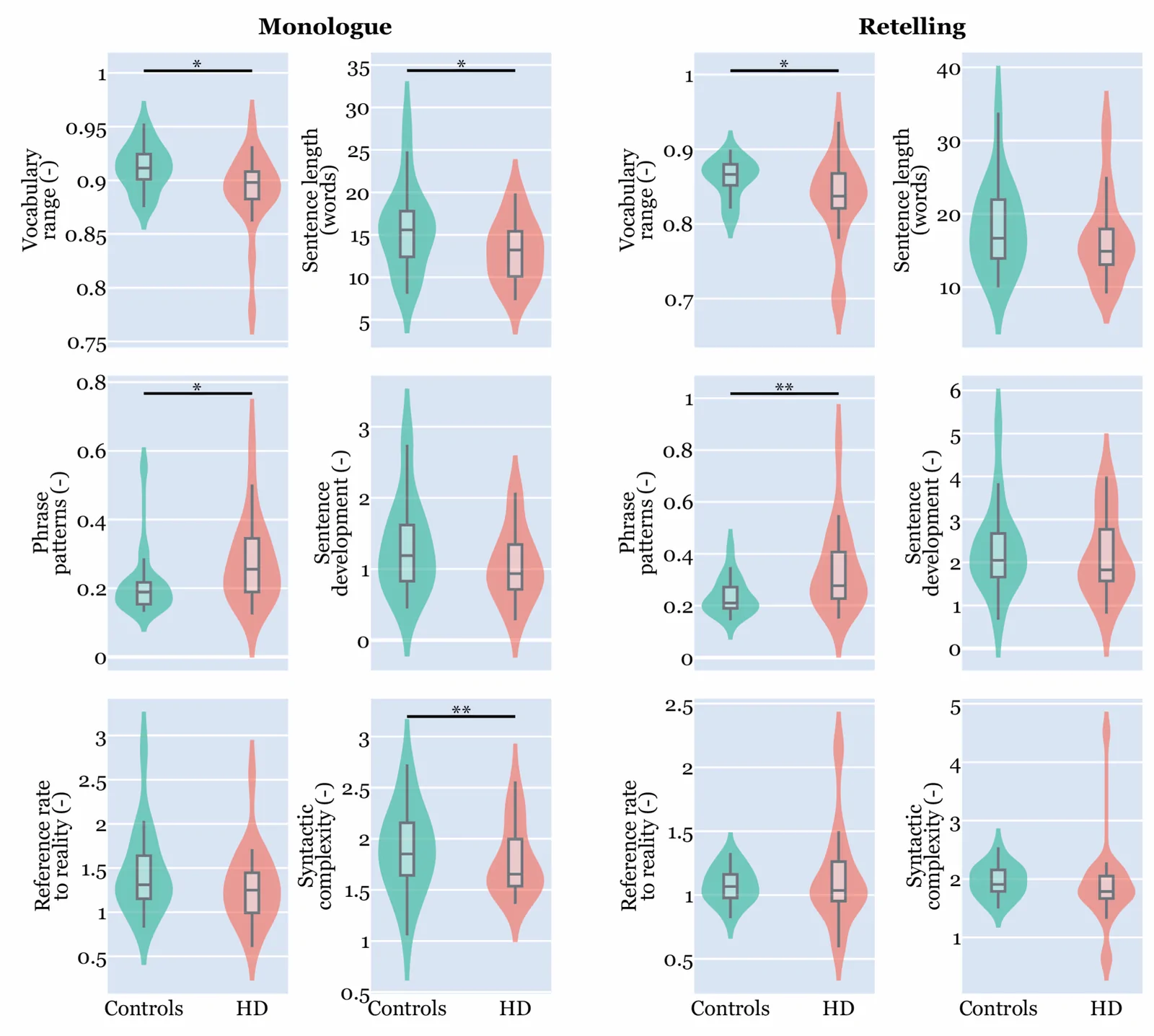
Group differences in linguistic features between healthy controls and Huntington’s disease. Violin plots depict the kernel density of the data, overlaid with boxplots, which represent the interquartile range (IQR; 25th–75th percentile), with the horizontal line indicating the median. Whiskers extend to the most extreme values within 1.5×IQR. Group differences were assessed using ANCOVA adjusted for age, sex, and language as covariates. Asterisks indicate statistical significance (*p < 0.05, **p < 0.01). HD = Huntington’s disease

Based on the monologue task, HD significantly differed from healthy controls in vocabulary range (F(1) = 5.28, q = 0.039), phrase patterns (F(1) = 6.25, q = 0.036), sentence length (F(1) = 6.024, q = 0.036), and syntactic complexity (F(1) = 9.13, q = 0.024). Based on the retelling task, HD differed from healthy controls in vocabulary range (F(1) = 6.55, q = 0.042) and phrase patterns (F(1) = 11.22, q = 0.010) (Fig. 3).

## Discussion

This study demonstrates that fully automated linguistic analysis of speech recorded unsupervised at home using a smartphone can capture clinically relevant markers in HD. Language characteristics derived from short speech tasks predicted core clinical outcomes, including cognitive performance, motor impairment, and functional capacity, with explained variances reaching up to 63%. These findings suggest that digital language metrics may serve as robust biomarkers for remote assessment of disease severity, capturing information typically reflected in gold-standard clinical scales while also providing complementary, non-overlapping markers related to higher-order language production. A median of 6 days for monologue and 3 days for retelling was sufficient to reach 90% of maximal predictive performance across investigated metrics. HD patients exhibited significant linguistic abnormalities in both the lexical and syntactic domain with consistent results across Czech and German speakers.

The prediction of UHDRS-TMS, a measure of motor decline, yielded the highest model predictive performance (R^2^ up to 0.63), with both lexical and syntactic features contributing, with syntactic features being particularly informative in the monologue task. This aligns with prior evidence that syntactic simplification, including reduced sentence length and complexity, is closely associated with motor impairments, likely due to neostriatal degeneration affecting motor-language coupling and grammatical production (Gordon and Illes 1987). Additionally, reduced production of complex sentences has been linked to motor speech deficits in HD (Murray 2000). Similarly, UHDRS-TFC, a measure of daily functioning, was predicted using both lexical and syntactic features. A previous study assessing speech production features, including temporal and rhythmic elements intertwined with syntax, has shown that these features predict TFC scores in HD, underscoring language as an indicator of overall disease burden (Riad et al. 2022). Measures of cognitive processing speed and executive function (SDMT, VF, ST, TMT-A and B) achieved prediction variances up to 57%. This aligns with previous findings showing that lower lexical diversity reflected broader cognitive decline, as patients increasingly rely on simpler words and reduced word variety to compensate for processing limitations (Gumus et al. 2024), whereas reduced fluency is linked to impaired lexical retrieval and executive function reflecting frontostriatal dysfunction (Ho et al. 2002). Similarly, shorter and simpler sentences may indicate slowed information processing, limiting the construction of syntactically complex utterances, a phenomenon previously observed in Parkinson’s disease (Grossman et al. 2002). In contrast, linguistic features did not achieve relevant model predictive performance for MMSE, likely due to its low sensitivity to subtle cognitive deficits in HD (Toh et al. 2014; Ringkøbing et al. 2020; Horta-Barba et al. 2023). Finally, language metrics were able, at least to some extent, to predict CAG repeat length, suggesting that the severity of language alterations is linked to a core biological marker of disease progression and supporting their potential utility as digital biomarkers for monitoring disease progression and treatment efficacy in future clinical trials. In addition, inclusion of UHDRS-DCL, a clinician-rated measure of confidence that motor signs are attributable to HD, did not diminish the contribution of linguistic features. Language measures continued to improve predictive performance beyond models including DCL for the majority of clinical outcomes, indicating that they capture complementary variance not explained by motor diagnostic confidence alone.

Group comparisons further confirmed that individuals with HD exhibited significant impairments in both lexical and syntactic language domains compared with healthy controls. Specifically, HD participants used a narrower vocabulary, produced shorter sentences, repeated the same phrase more often, and exhibited reduced syntactic complexity compared to controls. These findings align with previous studies reporting lexical decline, including naming and lexical retrieval difficulties (Caine et al. 1986; Gordon and Illes 1987; Illes 1989), phrase repetition (Tovar et al. 2020), as well as syntactic abnormalities such as shorter and simpler sentences (Murray 2000). These findings should be viewed in the context of heterogeneous disease stages within the HD cohort, with an unclear distribution of linguistic abnormalities across stages. Importantly, no significant interaction between group and the two examined languages was observed, suggesting that the linguistic alterations identified here are not language-specific but rather represent markers of disease-related pathology.

Comparison of the two speech tasks revealed complementary strengths. The retelling task demonstrated greater stability across days, requiring fewer recordings to reach the stability threshold in 9 out of 11 evaluated clinical scales. This likely reflects the higher degree of standardization in retelling, as participants repeat a story they have just heard, reducing variability and providing more consistent measurements across sessions; a median of 3 days was sufficient to reach 90% of maximum model predictive performance. In contrast, the monologue task elicited more spontaneous speech, which may better capture naturalistic language patterns. Notably, monologue speech provided superior differentiation between HD and healthy controls for syntactic features, including sentence length and complexity. This likely reflects the higher cognitive demands of generating original content, requiring participants to formulate ideas, organize them into coherent sentences, and retrieve relevant autobiographical memories. Interestingly, prediction of Trail Making Test was up to 3 times higher with monologue than retelling (R^2^ up to 0.57 vs. 0.18), consistent with the task’s greater engagement of planning and executive functions, as supported by prior research linking Trail Making performance to fluency-related linguistic features (Tovar et al. 2020). Due to variability in topics and the unstructured nature of monologue content, individual recordings were less consistent. Still, predictive performance improved when averaging across multiple sessions, reaching 90% of maximum model performance after 6 days of recording for most clinical outcomes. Collectively, these results underscore the importance of task selection in speech-based assessments. Retelling offers standardized, reliable measures, whereas monologue captures additional dimensions of linguistic impairment, particularly in syntactic organization and executive function demands.

Several limitations should be acknowledged. This pilot study included a cohort of 30 HD patients, and thus validation in larger cohorts is necessary to confirm and generalize these findings. Furthermore, the sample size of German participants was modest, which may have limited statistical power to detect language-related effects on group differences. However, the impact of language on group-level differences was only a secondary outcome, and previous research supports the notion that linguistic analysis is largely language-independent (Šubert et al. 2024). Inclusion of multiple disease stages may have increased variability in linguistic measures, and larger, stage-specific cohorts are needed to establish stage dependence and early detectability. All participants used the same smartphone model to ensure consistent audio quality in this proof-of-concept study. Future research should evaluate recordings collected across different smartphone types, although linguistic analyses are expected to remain robust to hardware variability and potential transcription errors (Fager and Burnfield 2015; Subert et al. 2025a).

In conclusion, our preliminary results suggest that smartphone-based, fully automated linguistic analysis may predict key clinical markers and differentiate HD from healthy controls consistently across two languages. These findings indicate that remote, language-based monitoring could serve as a low-burden complement to traditional clinical assessments. Further validation in larger cohorts is now required before establishing utility as a digital biomarker for longitudinal disease tracking and therapeutic evaluation.

## Supplementary Information

Below is the link to the electronic supplementary material.

Supplementary Material 1

## Data Availability

Individual participant data underlying the findings of this study are available from the corresponding author upon reasonable request. The data are not publicly available because they contain information that could compromise the privacy of study participants.
