# Correction: Remote smartphone-based spoken language screening predicts clinical markers in Huntington’s disease

**DOI:** 10.1007/s00702-026-03261-6

**Published:** 2026-09-28

**Authors:** Martin Šubert, Tomáš Kouba, Tereza Tykalová, Olga Klempířová, Alzbeta Mühlbäck, Jiří Klempíř, Jan Rusz

**Affiliations:** 1 Department of Circuit Theory, Faculty of Electrical Engineering, Czech Technical University in Prague, Technická 2, Praha 6, 160 00 Czech Republic; 2 Department of Neurology, Centre of Clinical Neuroscience, First Faculty of Medicine, Charles University and General University Hospital, Prague, Czech Republic; 3 Huntington Center South, kbo-isar-Amper-Klinikum, Taufkirchen (Vils), Germany; 4 Department of Neurology, University Hospital Ulm, Ulm University, Ulm, Germany

**Correction: Journal of Neural Transmission**

**https://doi.org/10.1007/s00702-026-03173-5**

In the original version of this article, grant dedication to the Funding (Czech Ministry of Health (grant nr. MH CZ–DRO-VFN64165)) was missing in the funding section.

Funding section, which previously read as

Open access publishing supported by the institutions participating in the CzechELib Transformative Agreement. This work was supported by the EU Joint Programme – Neurodegenerative Disease Research (JPND) project “EXPAND-RED”, with funding from Czech Ministry of Education, Youth and Sports under the JPND framework (project ID 9F25002), and by the Czech Ministry of Health (grant nr. NW25-04–00052). This work is generated within the European Reference Network for Rare Neurological Diseases—Project ID No 739510.

but should have read

Open access publishing supported by the institutions participating in the CzechELib Transformative Agreement. This work was supported by the EU Joint Programme – Neurodegenerative Disease Research (JPND) project “EXPAND-RED”, with funding from Czech Ministry of Education, Youth and Sports under the JPND framework (project ID 9F25002), and by the Czech Ministry of Health (grants nr. NW25-04-00052 and MH CZ–DRO-VFN64165). This work is generated within the European Reference Network for Rare Neurological Diseases—Project ID No 739510. 

