# Supplementary material for: Remote smartphone-based spoken language screening predicts clinical markers in Huntington’s disease
Source: J Neural Transm (Vienna). 2026 May 14;133(9):2479–90. doi: 10.1007/s00702-026-03173-5 (PMC13687125; doi:10.1007/s00702-026-03173-5)
Supplement: Supplementary file 1 [file 702_2026_3173_MOESM1_ESM.pdf]

**Table S1.** Description of monologue speech task

| Thematic category | Prompt (English equivalent) |
|---|---|
| Description of the previous day | *"Try to describe your day yesterday in as much detail as possible."* |
| Favorite places/food/activities | *"Describe the town/village where you live. What interesting places nearby would you recommend to someone visiting from another region?"* |
| | *"Describe the town/village where you spent your childhood. What interesting places nearby would you recommend to someone visiting from another region?"* |
| | *"What was your most interesting summer vacation? What was the itinerary, and what interesting places did you visit?"* |
| | *"What is your favorite food? How often and where do you eat it? Who prepares it for you? Which local restaurant would you recommend?"* |
| | *"Describe your favorite TV show/series. What type of movies do you like to watch?"* |
| | *"What is your favorite hobby? What do you like to do in your free time?"* |
| | *"Which school did you attend in the past? Which school subjects did you enjoy or dislike? Which classmates do you fondly remember?"* |
| Hypothetical reasoning, imagination, and autobiographical memory | *"What was the happiest moment in your life?"* |
| | *"What was the silliest thing you have ever done?"* |
| | *"What is your biggest bad habit?"* |
| | *"Which person in your life has influenced you the most, and why?"* |
| | *"If you could be an animal, which one would it be and why?"* |
| | *"If you won 20 million in the lottery, how would you use the winnings?"* |
| | *"If you could live in another historical era, which one would it be and why?"* |

Each session included three questions. Each question was chosen from one thematic category.

**Table S2.** Description of retelling speech task

| Language | Fairy tale (English equivalent) | Number of words in the reference fairy tale |
|---|---|---|
| Czech | *Hansel and Gretel* | 269 |
| | *Sleeping Beauty* | 225 |
| | *The Emperor's New Clothes* | 235 |
| | *Snow White and the Seven Dwarfs* | 258 |
| | *Cinderella* | 282 |
| | *The Three Billy Goats Gruff* | 262 |
| | *Little Red Riding Good* | 267 |
| German | *Hansel and Gretel* | 210 |
| | *Sleeping Beauty* | 223 |
| | *The Emperor's New Clothes* | 228 |
| | *Rumpelstiltskin* | 276 |
| | *The Bremen Town Musicians* | 221 |
| | *The Frog King* | 261 |
| | *Rapunzel* | 264 |

Each session included one fairy tale.

**Table S3** Predictive performance of linguistic features for clinical markers with adjustment for Diagnostic Confidence Level variable as predictor in monologue tasks

| Clinical scale | Predictors selected through feature selection (β, p-value) | $R^2$ | $\Delta R^2$ | p-value |
|---|---|---|---|---|
| UHDRS-TMS | Syntactic complexity (β=-0.61, p<0.001)<br>Czech/German language (β=0.73, p<0.001)<br>DCL (β=0.53, p<0.001) | 0.76 | 0.18 | <0.001 |
| UHDRS-TFC | Vocabulary range (β=0.42, p<0.001)<br>Phrase patterns (β=0.36, p=0.0071)<br>Sentence length (β=0.41, p=0.0057)<br>DCL (β=-0.50, p=0.0021) | 0.63 | 0.25 | <0.001 |
| SDMT | Sentence length (β=1.08, p<0.001)<br>Sentence development (β=-0.89, p<0.001)<br>Czech/German language (β=-0.36, p=0.0083)<br>DCL (β=-0.43, p=0.0021) | 0.62 | 0.22 | <0.001 |
| Lexical verbal fluency | Phrase patterns (β=-0.20, p=0.19)<br>Sentence length (β=1.50, p<0.001)<br>Sentence development (β=-0.82, p=0.010)<br>Syntactic complexity (β=-0.86, p<0.001) | 0.41 | 0.24 | <0.001 |
| Categorical verbal fluency | Sentence length (β=0.90, p<0.001)<br>Sentence development (β=-0.45, p=0.044)<br>Czech/German language (β=-0.29, p=0.035)<br>DCL (β=-0.37, p=0.0094) | 0.61 | 0.19 | <0.001 |
| Stroop Color Test | Vocabulary range (β=0.35, p=0.020)<br>Reference rate to reality (β=-0.30, p=0.045)<br>Sentence length (β=0.39, p=0.011)<br>DCL (β=-0.41, p=0.0090) | 0.54 | 0.17 | <0.001 |
| Stroop Word Test | Vocabulary range (β=0.14, p=0.29)<br>Sentence length (β=1.02, p<0.001)<br>Sentence development (β=-0.70, p=0.0052)<br>DCL (β=-0.25, p=0.12) | 0.50 | 0.30 | <0.001 |
| Stroop Interference Test | Vocabulary range (β=0.15, p=0.32)<br>Sentence length (β=0.62, p=0.030)<br>Sentence development (β=-0.37, p=0.17)<br>DCL (β=-0.41, p=0.026) | 0.37 | 0.08 | 0.002 |
| Trail Making Test A | Phrase patterns (β=0.35, p=0.0094)<br>Sentence length (β=-1.29, p<0.001)<br>Sentence development (β=1.13, p<0.001) | 0.56 | 0.43 | <0.001 |
| Trail Making Test B | Phrase patterns (β=0.32, p=0.031)<br>Sentence length (β=-0.93, p=0.013)<br>Sentence development (β=0.96, p=0.0031)<br>Syntactic complexity (β=-0.23, p=0.25) | 0.45 | 0.17 | <0.001 |
| CAG | Sentence length (β=-0.24, p=0.16)<br>Age (β=-0.49, p=0.0015)<br>DCL (β=0.35, p=0.040) | 0.38 | 0.004 | 0.002 |

β = standardized beta value of individual predictors, *$\Delta R^2$ = increase in $R^2$ from the model including only demographic variables (age, sex, and Czech/German language) and DCL, and the model additionally including linguistic features, DCL = Diagnostic Confidence Level, UHDRS-TMS = Unified Huntington's Disease Rating Scale Total Motor Score, UHDRS-TFC = Unified Huntington's Disease Rating* Scale Total Functional Capacity, SDMT = Symbol Digit Modalities Test, CAG = cytosine-adenine-guanine.

**Table S4** Predictive performance of linguistic features for clinical markers with adjustment for Diagnostic Confidence Level variable as predictor in retelling tasks

| Clinical scale | Predictors selected through feature selection (β, p-value) | $R^2$ | $\Delta R^2$ | p-value |
|---|---|---|---|---|
| UHDRS-TMS | Vocabulary range (β=-0.20, p=0.047)<br>Phrase patterns (β=0.45, p<0.001)<br>DCL (β=0.52, p<0.001) | 0.81 | 0.23 | <0.001 |
| UHDRS-TFC | Vocabulary range (β=0.16, p=0.18)<br>Phrase patterns (β=-0.52, p<0.001)<br>Reference rate to reality (β=0.30, p=0.0040)<br>DCL (β=-0.47, p<0.001) | 0.71 | 0.33 | <0.001 |
| SDMT | Phrase patterns (β=-0.52, p<0.001)<br>Sentence development (β=-0.27, p=0.031)<br>Syntactic complexity (β=0.12, p=0.27)<br>DCL (β=-0.61, p<0.001) | 0.65 | 0.25 | <0.001 |
| Lexical verbal fluency | Vocabulary range (β=0.60, p<0.001)<br>Sentence development (β=0.25, p=0.12) | 0.33 | 0.16 | <0.001 |
| Categorical verbal fluency | Vocabulary range (β=0.36, p=0.0073)<br>Sentence development (β=0.21, p=0.094)<br>Syntactic complexity (β=0.23, p=0.053)<br>DCL (β=-0.47, p=0.0011) | 0.62 | 0.20 | <0.001 |
| Stroop Color Test | Phrase patterns (β=-0.57, p<0.001)<br>Reference rate to reality (β=0.27, p=0.035)<br>DCL (β=-0.47, p<0.001) | 0.60 | 0.23 | <0.001 |
| Stroop Word Test | Phrase patterns (β=-0.77, p<0.001)<br>Reference rate to reality (β=0.30, p=0.018)<br>Language (β=0.33, p=0.0081)<br>DCL (β=-0.29, p=0.019) | 0.59 | 0.40 | <0.001 |
| Stroop Interference Test | Phrase patterns (β=-0.66, p<0.001)<br>Reference rate to reality (β=0.30, p=0.051)<br>Age (β=-0.22, p=0.12)<br>DCL (β=-0.39, p=0.0067) | 0.53 | 0.24 | 0.002 |
| Trail Making Test A | Phrase patterns (β=0.49, p=0.013)<br>Reference rate to reality (β=-0.29, p=0.091)<br>Language (β=-0.47, p=0.0080)<br>DCL (β=0.32, p=0.058) | 0.26 | 0.13 | <0.001 |
| Trail Making Test B | Language (β=-0.50, p=0.0037)<br>DCL (β=0.44, p=0.0096) | 0.27 | 0 | <0.001 |
| CAG | Vocabulary range (β=-0.14, p=0.41)<br>Phrase patterns (β=0.29, p=0.072)<br>Age (β=-0.42, p=0.0086)<br>DCL (β=0.31, p=0.049) | 0.44 | 0.06 | 0.002 |

β = standardized beta value of individual predictors, *$\Delta R^2$ = increase in $R^2$ from the model including only demographic variables (age, sex, and Czech/German language) and DCL, and the model additionally including linguistic features, DCL = Diagnostic Confidence Level, UHDRS-TMS = Unified Huntington's Disease Rating Scale Total Motor Score, UHDRS-TFC = Unified Huntington's Disease Ratin*g Scale Total Functional Capacity, SDMT = Symbol Digit Modalities Test, CAG = cytosine-adenine-guanine.

**Figure S1.** Normative data of language characteristics across two languages for monologue and retelling tasks

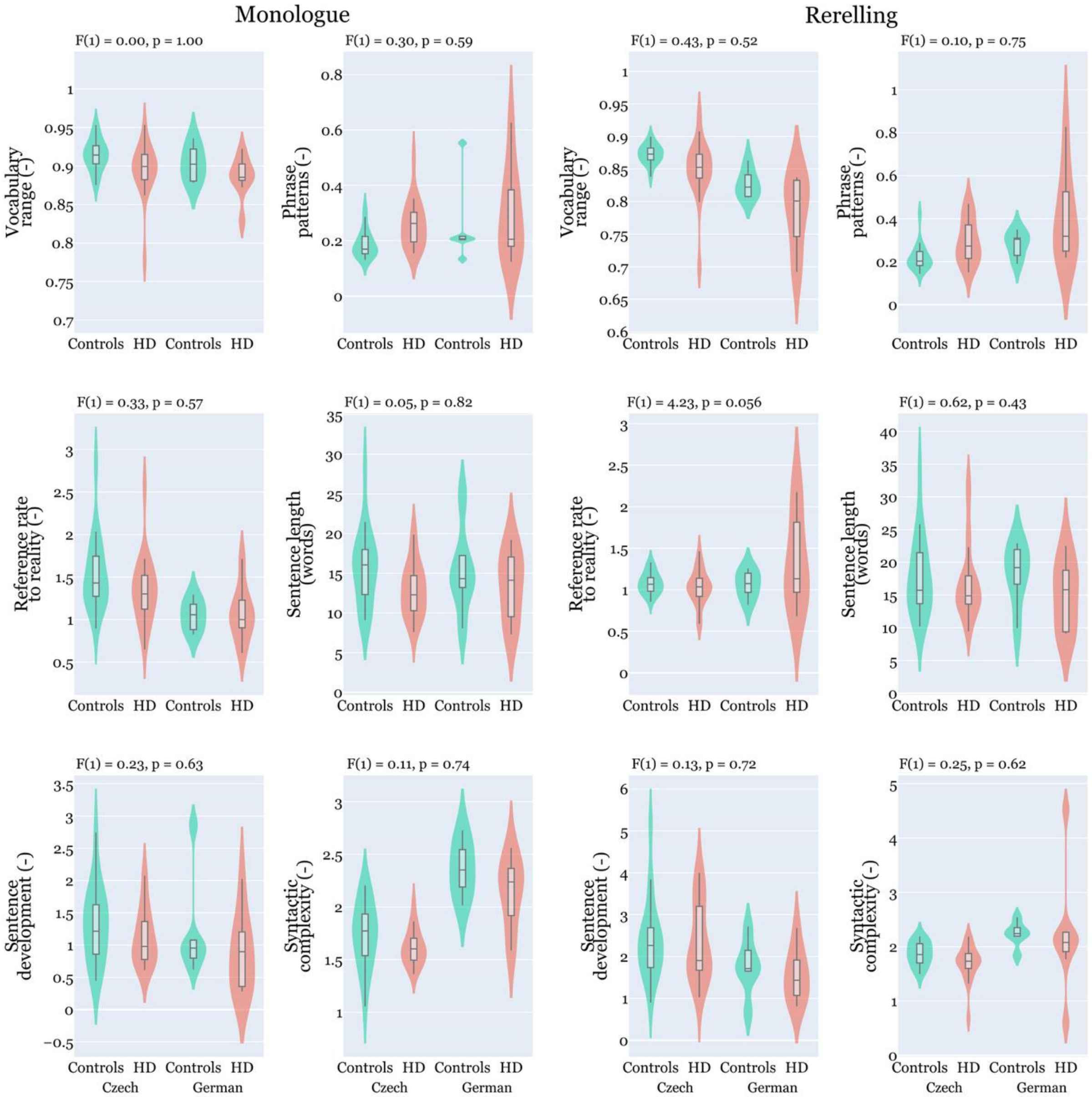


Violin plots depict the kernel density of the data, overlaid with boxplots, which represent the interquartile range (IQR; 25th–75th percentile), with the horizontal line indicating the median. Whiskers extend to the most extreme values within 1.5×IQR. Results above each panel report the group per language interaction based on analysis of covariance, with age and sex as covariates. HD = Huntington's disease.
